# A comprehensive dataset on pollinator diversity, visitation rates, individual-based traits, and pollination success across four plant species in an urban garden experiment in Zurich, Switzerland

**DOI:** 10.1016/j.dib.2025.112013

**Published:** 2025-08-29

**Authors:** Merin Reji Chacko, Marco Moretti, David Johannes Frey

**Affiliations:** a Biodiversity and Conservation Biology, Swiss Federal Research Institute WSL, Zürcherstrasse 111, 8903 Birmensdorf, Switzerland; b Museo Cantonale di Storia Naturale, Viale Carlo Cattaneo 4, 6900 Lugano, Switzerland

**Keywords:** Functional traits, Densification, Ecosystem services, Phytometer, Reproductive success, Urbanisation gradient

## Abstract

This dataset presents flower visitation frequency, pollinator richness, and direct measures of pollination success for four focal plant species from a field experiment in 24 home gardens in the city of Zurich, Switzerland. The home gardens were selected to vary independently in local flowering species richness and the proportion of impervious surface in a 500-m radius around the garden, a common proxy for urban intensity and associated habitat loss. We used a phytometer species approach with the following four insect-pollinated plant species: wild carrot (*Daucus carota L.*), radish (*Raphanus sativus* L.), common sainfoin (*Onobrychis viciifolia* Scop.) and common comfrey (*Symphytum officinale* L.).

We provide the species richness and hourly visitation frequency of 167 flower visitor taxa across multiple taxonomic groups (bees, wasps, beetles, and hoverflies) from multiple sampling dates across the full flowering period of the phytometer species. We collected and identified 5,794 individuals, of which the vast majority (99.5%) were identified to the species or genus level. We provide several functional trait measurements at the individual level. For bees, we measured intertegular distance and proboscis length (the combined lengths of prementum and glossa); for the other taxa, we measured forewing length and the lengths of the labellum, prementum, and fulcrum. We additionally provide seed and/or fruit set, a direct measure of reproductive success for all phytometer plants.

Further datasets for these gardens exist, linking soil and soil arthropod diversity data, bird predation data, and plant diversity and properties sampled during the same period. This dataset enables further investigations into the composition of novel anthropogenic pollinator communities, such as analyses comparing multiple cities. The fine temporal resolution of flower visitor frequency additionally provides the opportunity to conduct time series analyses of diurnal pollinator communities across environmental gradients.

Specifications Table

**Table:** 

Subject	Biology
Specific subject area	*Urban ecology*
Type of data	Raw and aggregated Excel sheet with raw sampling data and meta-data CSV files for cleaned data PDF files of sampling instructions and sampling form (original German and English translation) R scripts reproducing the figures in this present dataset
Data collection	Flower visitation frequency was collected by pre-trained volunteer scientists conducting a field survey using a prepared survey sheet. We collected insects using a 50 mm by 100 mm polypropylene beaker with a foam plug (Semadeni AG, Ostermundigen), which were then identified by taxonomic experts. We measured bee traits using the Olympus SZX12 Microscope and Olympus image analysis software (Version 510; Olympus Soft Imaging Solutions GmbH). For the other insects, we took microphotographs with a Leica stereo microscope. The labellum-prementum ratio was calculated by dividing the labellum by the prementum value. Fruit/seed sets were counted manually.
Data source location	24 home garden sites in the city of Zurich, Switzerland (47°22’N, 8°33’E).
Data accessibility	Repository name: EnviDat Data identification number: 10.16904/envidat.676 Direct URL to data: https://www.doi.org/10.16904/envidat.676
Related research article	Casanelles-Abella, Joan, Simone Fontana, Bertrand Fournier, David Frey, and Marco Moretti. 2023. “Low Resource Availability Drives Feeding Niche Partitioning between Wild Bees and Honeybees in a European City.” Ecological Applications 33(1): e2727. https://doi.org/10.1002/eap.2727 [1]

## 1 Value of the Data

• The data include individual flower visitor records with hourly visitation records capturing both abundance (hourly visitation frequency) and taxonomic richness across four major pollinator groups: bees, hoverflies, wasps, and beetles. These data were collected from the flowers of four phytometer plant species grown in standardised pot arrays. The phytometer plants were selected to span a gradient of flower visitor specificity. These standardised and high-resolution data offer unique insights into diurnal foraging patterns across taxa and flower types based on a quasi-orthogonal experimental design: two independent gradients of landscape-scale urban intensification (increasing amount of impervious surface) and local-scale floral richness.
• This dataset provides not only a common proxy for pollination success (flower visitation frequency), but also direct measures of reproductive success (fruit and seed set), enabling a quantitative evaluation of pollination services across both plant and pollinator functional groups.
• The dataset offers individual-level trait data (e.g. intertegular distance, forewing span, and tongue morphology) for 167 taxa. These measurements can support analyses of trait-matching, functional diversity, or mobility in pollinator communities within fragmented urban landscapes.
• The data are particularly valuable for assessing the contribution of non-bee pollinators to pollination services, such as hoverflies, beetles and wasps, often-overlooked groups despite emerging evidence of their ecological importance. Indeed, non-bee pollinators can account for up to 40% of flower visits globally [2] and wasps have even been demonstrated to provide comparable pollination services to bees [3].
• Because the experimental data were collected along a landscape-scale gradient of urban intensification, they enable comparative studies on how urban land use and cover affects plant–pollinator interactions, trait filtering, and the provisioning of pollination services.
• The dataset is compatible with other existing datasets from the same experimental gardens (covering above and belowground plant [4] and vertebrate [5] communities, soil properties and management intensity [6]) and can therefore be used for multi-trophic studies of biodiversity and ecosystem functioning in urban environments.

## 2 Background

This dataset was collected as part of the BetterGardens (https://www.bettergardens.ch/en/) project, which investigates how biodiversity, soil quality, and ecosystem services respond to local and landscape-scale variation in urban gardens in Zurich, and other cities in Switzerland. The project combines ecological and social research to understand how gardeners make decisions and how these choices affect biodiversity, ecosystem services and well-being. Here, we aimed to examine how densification in cities shapes pollinator communities and their ability to pollinate plants with varying flower morphologies. Therefore, we selected 24 home gardens spanning independent gradients of local, garden-level flowering plant species richness and landscape-scale urban intensity, measured by the relative proportion of impervious surface. A widely used method in pollination studies to assess pollination services, plant reproductive success and environmental effects on pollination is the phytometer approach, which uses standardised, potted insect-pollinated self-incompatible plants as indicators [[7], [8], [9]]. Using the phytometer approach, we installed experimental arrays of pots of four insect-pollinated plant species that differ in floral morphology and visitor specificity.

Pollinator communities—including bees, hoverflies, wasps, and beetles—were sampled by pre-trained volunteers during nine-hour observation periods across the flowering season. We obtained hourly flower visitation frequencies at high taxonomic resolution and collected 5,794 individuals for trait measurements. At the end of the flowering period, we measured fruit and seed sets as direct indicators of pollination success.

In addition to the published article [1], this dataset offers a finer temporal resolution and includes raw individual-level trait measurements. It enables future investigations into diurnal patterns of pollinator activity, trait–functioning relationships, and quantitative species interaction networks. The dataset also facilitates comparisons of community composition and pollination success to other cities, such as Lausanne and Berne, for which other BetterGardens data were collected.

## 3 Data Description

We provide data collected from a field study investigating the diversity and flower visitation frequency of pollinators and subsequent effects on pollination success across 24 home gardens in Zurich, Switzerland. The data are organised into structured folders, each containing specific files related to garden site locations, taxonomic information, sampling protocols, field data, trait measurements, and pollination success outcomes. An overview of the files, their location within the repository, file types, contents, and number of records is provided in Table 1. The data are openly available in the EnviDat repository [10], which includes the following directories:

• metadata files (01_metadata/), providing a comprehensive list of all variables included in each tabular dataset (data_description.xlsx) and a general repository summary (README.txt),
• sampling protocols (02_sampling_protocol/), containing the original field sampling instructions in German (protocol_german.pdf) and the English translation (protocol_english.pdf),
• garden site coordinates (03_site_data/), listing the geographic coordinates (latitude, longitude) for the 24 home gardens included in the study (garden_site_coordinates.csv), and the garden ID number, which was standardised across all data from the Better Gardens project, such that other data can be seamlessly combined.
• taxonomic checklist (04_taxonomic_data/), containing all recorded taxa (see Table 2) identified in the sampling, including taxonomic rank, order, family, genus, and species (taxa_checklist.csv),

**Table 2:** List of observed taxa, the number of gardens they have occurred in and number of observations with nectar robbery.

Pollinator group	Family	Taxon	Taxonomic rank	Total observations	Number of gardens	Number of observations with nectar robbery or illegitimate visitors
Bees	Andrenidae	*Andrena agilissima*	Species	1	1	0
Bees	Andrenidae	*Andrena bicolor*	Species	10	7	0
Bees	Andrenidae	*Andrena chrysosceles*	Species	3	3	0
Bees	Andrenidae	*Andrena dorsata*	Species	1	1	0
Bees	Andrenidae	*Andrena minutula*	Species	42	13	0
Bees	Andrenidae	*Andrena minutuloides*	Species	5	3	0
Bees	Andrenidae	*Andrena ovatula*	Species	1	1	0
Bees	Andrenidae	*Andrena subopaca*	Species	13	3	0
Bees	Anthophorinae	*Ceratina cyanea*	Species	1	1	1
Bees	Anthophorinae	*Eucera nigrescens*	Species	1	1	1
Bees	Apidae	*Apis mellifera*	Species	577	24	68
Bees	Apidae	*Bombus hortorum*	Species	42	12	0
Bees	Apidae	*Bombus humilis*	Species	5	4	0
Bees	Apidae	*Bombus hypnorum*	Species	2	2	1
Bees	Apidae	*Bombus lapidarius*	Species	14	8	0
Bees	Apidae	*Bombus pascuorum*	Species	235	24	0
Bees	Apidae	*Bombus pratorum*	Species	10	8	0
Bees	Apidae	*Bombus* sp.	Genus	3	3	0
Bees	Apidae	*Bombus terrestris-complex*	Species complex	57	22	7
Bees	Apidae	*Bombus vestalis*	Species	2	1	0
Bees	Apidae	*Bombus wurflenii*	Species	1	1	0
Bees	Colletidae	*Colletes daviesanus*	Species	3	3	0
Bees	Colletidae	*Colletes similis*	Species	1	1	0
Bees	Colletidae	*Hylaeus brevicornis*	Species	5	3	0
Bees	Colletidae	*Hylaeus clypearis*	Species	3	3	0
Bees	Colletidae	*Hylaeus communis*	Species	210	22	1
Bees	Colletidae	*Hylaeus confusus*	Species	24	11	0
Bees	Colletidae	*Hylaeus cornutus*	Species	1	1	0
Bees	Colletidae	*Hylaeus difformis*	Species	4	4	0
Bees	Colletidae	*Hylaeus gibbus*	Species	1	1	0
Bees	Colletidae	*Hylaeus gredleri*	Species	79	15	0
Bees	Colletidae	*Hylaeus hyalinatus*	Species	85	19	0
Bees	Colletidae	*Hylaeus leptocephalus*	Species	10	4	0
Bees	Colletidae	*Hylaeus pictipes*	Species	98	15	1
Bees	Colletidae	*Hylaeus punctatus*	Species	222	22	0
Bees	Colletidae	*Hylaeus sinuatus*	Species	273	22	2
Bees	Colletidae	*Hylaeus* sp.	Genus	9	7	0
Bees	Colletidae	*Hylaeus styriacus*	Species	8	5	0
Bees	Colletidae	*Hylaeus taeniolatus*	Species	38	9	0
Bees	Halictidae	*Halictus simplex-complex*	Species complex	7	3	0
Bees	Halictidae	*Halictus tumulorum*	Species	42	16	12
Bees	Halictidae	*Lasioglossum calceatum*	Species	24	11	1
Bees	Halictidae	*Lasioglossum fulvicorne*	Species	1	1	0
Bees	Halictidae	*Lasioglossum glabriusculum*	Species	3	2	1
Bees	Halictidae	*Lasioglossum laticeps*	Species	460	23	8
Bees	Halictidae	*Lasioglossum lativentre*	Species	1	1	0
Bees	Halictidae	*Lasioglossum leucozonium*	Species	1	1	1
Bees	Halictidae	*Lasioglossum malachurum*	Species	29	8	0
Bees	Halictidae	*Lasioglossum morio*	Species	244	22	23
Bees	Halictidae	*Lasioglossum nitidulum*	Species	137	18	3
Bees	Halictidae	*Lasioglossum pauxillum*	Species	667	21	40
Bees	Halictidae	*Lasioglossum politum*	Species	3	2	0
Bees	Halictidae	*Lasioglossum villosulum*	Species	1	1	0
Bees	Halictidae	*Lasioglossum zonulum*	Species	1	1	1
Bees	Halictidae	*Sphecodes niger*	Species	2	2	0
Bees	Halictidae	*Sphecodes* sp.	Genus	2	1	0
Bees	Megachilidae	*Anthidium manicatum*	Species	9	7	4
Bees	Megachilidae	*Anthidium oblongatum*	Species	11	6	0
Bees	Megachilidae	*Anthidium punctatum*	Species	1	1	0
Bees	Megachilidae	*Anthidium strigatum*	Species	1	1	0
Bees	Megachilidae	*Chelostoma campanularum*	Species	2	2	1
Bees	Megachilidae	*Chelostoma rapunculi*	Species	8	8	7
Bees	Megachilidae	*Megachile centuncularis*	Species	1	1	0
Bees	Megachilidae	*Megachile ericetorum*	Species	16	10	2
Bees	Megachilidae	*Megachile willughbiella*	Species	21	10	10
Bees	Megachilidae	*Osmia adunca*	Species	2	2	1
Bees	Megachilidae	*Osmia bicornis*	Species	1	1	0
Bees	Megachilidae	*Osmia brevicornis*	Species	1	1	0
Bees	Megachilidae	*Osmia caerulescens*	Species	21	9	13
Bees	Megachilidae	*Osmia leucomelana*	Species	5	3	0
Beetles		Coleoptera	Order	5	3	0
Beetles	Buprestidae	*Anthaxia nitidula*	Species	12	9	0
Beetles	Cerambycidae	*Clytus arietis*	Species	2	2	0
Beetles	Cerambycidae	*Rutpela maculata*	Species	2	2	0
Beetles	Cerambycidae	*Stenurella melanura*	Species	1	1	0
Beetles	Cerambycidae	*Stictoleptura rubra*	Species	10	7	0
Beetles	Chrysomelidae	*Clytra laeviuscula*	Species	2	2	0
Beetles	Cleridae	*Trichodes alvearius*	Species	3	3	0
Beetles	Cleridae	*Trichodes apiarius*	Species	2	2	0
Beetles	Dasytidae	*Dasytes plumbeus*	Species	9	7	0
Beetles	Malachiidae	*Malachius bipustulatus*	Species	2	2	0
Beetles	Mordellidae	*Mordella* sp.	Genus	3	3	0
Beetles	Mordellidae	*Variimorda* sp.	Genus	21	11	0
Beetles	Oedemeridae	*Anogcodes rufiventris*	Species	33	13	0
Beetles	Oedemeridae	*Oedemera femorata*	Species	5	4	0
Beetles	Oedemeridae	*Oedemera lurida*	Species	4	4	0
Beetles	Oedemeridae	*Oedemera nobilis*	Species	10	3	0
Beetles	Scarabaeidae	*Hoplia philanthus*	Species	13	2	0
Beetles	Scarabaeidae	*Oxythyrea funesta*	Species	1	1	0
Beetles	Scarabaeidae	*Trichius fasciatus*	Species	17	10	0
Hoverflies		Diptera	Order	25	13	0
Hoverflies	Syrphidae	*Cheilosia* sp.	Genus	89	14	0
Hoverflies	Syrphidae	*Chrysogaster solstitialis*	Species	2	2	0
Hoverflies	Syrphidae	*Chrysotoxum intermedium*	Species	1	1	1
Hoverflies	Syrphidae	*Chrysotoxum vernale*	Species	1	1	0
Hoverflies	Syrphidae	*Epistrophe melanostoma*	Species	1	1	0
Hoverflies	Syrphidae	*Episyrphus balteatus*	Species	527	24	21
Hoverflies	Syrphidae	*Eristalis arbustorum*	Species	127	19	1
Hoverflies	Syrphidae	*Eristalis interrupta*	Species	7	6	0
Hoverflies	Syrphidae	*Eristalis pertinax*	Species	2	1	0
Hoverflies	Syrphidae	*Eristalis tenax*	Species	28	13	0
Hoverflies	Syrphidae	*Eupeodes corollae*	Species	164	22	2
Hoverflies	Syrphidae	*Eupeodes latilunulatus*	Species	1	1	0
Hoverflies	Syrphidae	*Eupeodes luniger*	Species	2	2	0
Hoverflies	Syrphidae	*Helophilus pendulus*	Species	2	2	0
Hoverflies	Syrphidae	*Melangyna auricollis*	Species	3	1	0
Hoverflies	Syrphidae	*Melangyna umbellatarum*	Species	1	1	0
Hoverflies	Syrphidae	*Melanostoma mellinum*	Species	34	16	0
Hoverflies	Syrphidae	*Melanostoma scalare*	Species	11	10	1
Hoverflies	Syrphidae	*Meliscaeva auricollis*	Species	1	1	0
Hoverflies	Syrphidae	*Myathropa florea*	Species	8	3	0
Hoverflies	Syrphidae	*Orthonevra nobilis*	Species	2	2	0
Hoverflies	Syrphidae	*Paragus albifrons*	Species	1	1	0
Hoverflies	Syrphidae	*Paragus haemorrhous*	Species	1	1	0
Hoverflies	Syrphidae	*Paragus* sp.	Genus	9	7	0
Hoverflies	Syrphidae	*Pipiza* sp.	Genus	2	1	0
Hoverflies	Syrphidae	*Pipizella* sp.	Genus	41	12	0
Hoverflies	Syrphidae	*Pipizella viduata*	Species	85	16	0
Hoverflies	Syrphidae	*Pipizella virens*	Species	1	1	0
Hoverflies	Syrphidae	*Platycheirus albimanus*	Species	16	7	0
Hoverflies	Syrphidae	*Platycheirus scutatus*	Species	1	1	0
Hoverflies	Syrphidae	*Platycheirus* sp.	Genus	1	1	1
Hoverflies	Syrphidae	*Scaeva pyrastri*	Species	6	6	0
Hoverflies	Syrphidae	*Scaeva selenitica*	Species	1	1	0
Hoverflies	Syrphidae	*Sphaerophoria interrupta*	Species	1	1	0
Hoverflies	Syrphidae	*Sphaerophoria scripta*	Species	184	21	3
Hoverflies	Syrphidae	*Sphaerophoria* sp.	Genus	224	21	9
Hoverflies	Syrphidae	*Sphaerophoria taeniata*	Species	1	1	0
Hoverflies	Syrphidae	*Syritta pipiens*	Species	45	17	1
Hoverflies	Syrphidae	*Syrphidae*	Species	2	2	0
Hoverflies	Syrphidae	*Syrphus ribesii*	Species	7	7	0
Hoverflies	Syrphidae	*Syrphus torvus*	Species	1	1	0
Hoverflies	Syrphidae	*Syrphus vitripennis*	Species	4	3	0
Hoverflies	Syrphidae	*Volucella zonaria*	Species	1	1	0
Wasps	Chrysididae	Chrysididae	Species	1	1	0
Wasps	Chrysididae	*Chrysis gracillima*	Species	3	3	0
Wasps	Chrysididae	*Chrysis viridula*	Species	1	1	0
Wasps	Chrysididae	*Hedychrum gerstaeckeri*	Species	5	5	0
Wasps	Chrysididae	*Holopyga generosa*	Species	3	3	0
Wasps	Chrysididae	*Omalus biaccinctus*	Species	10	5	2
Wasps	Chrysididae	*Pseudomalus auratus*	Species	2	2	1
Wasps	Chrysididae	*Pseudomalus pusillus*	Species	1	1	0
Wasps	Crabronidae	*Cerceris quinquefasciata*	Species	5	1	0
Wasps	Crabronidae	*Cerceris rybyensis*	Species	14	6	0
Wasps	Crabronidae	*Ectemnius dives*	Species	4	4	0
Wasps	Crabronidae	*Ectemnius lituratus*	Species	1	1	0
Wasps	Crabronidae	*Ectemnius rubicola*	Species	1	1	0
Wasps	Crabronidae	*Gorytes quinquecinctus*	Species	15	5	0
Wasps	Crabronidae	*Lestica clypeata*	Species	2	2	0
Wasps	Crabronidae	*Oxybelus bipunctatus*	Species	2	2	0
Wasps	Crabronidae	*Oxybelus* sp.	Genus	1	1	0
Wasps	Crabronidae	*Oxybelus trispinosus*	Species	2	2	0
Wasps	Crabronidae	*Passaloceus* sp.	Genus	1	1	0
Wasps	Crabronidae	*Passaloecus borealis*	Species	3	3	1
Wasps	Crabronidae	*Psenulus pallipes*	Species	2	1	0
Wasps	Crabronidae	*Spilomena* sp.	Genus	1	1	0
Wasps	Crabronidae	*Trypoxylon minus*	Species	1	1	0
Wasps	Pompilidae	*Anoplius nigerrimus*	Species	6	6	0
Wasps	Pompilidae	*Arachnospila spissa*	Species	7	5	0
Wasps	Sapygidae	*Sapygina decemguttata*	Species	3	3	0
Wasps	Sphecidae	*Isodontia mexicana*	Species	1	1	0
Wasps	Sphecidae	*Mimumesa* sp.	Genus	1	1	0
Wasps	Vespidae	*Ancistrocerus claripennis*	Species	1	1	0
Wasps	Vespidae	*Ancistrocerus gazella*	Species	3	3	1
Wasps	Vespidae	*Dolichovespula saxonica*	Species	1	1	1
Wasps	Vespidae	*Polistes dominulus*	Species	33	17	0
Wasps	Vespidae	*Vespula vulgaris*	Species	1	1	0
• raw sampling data (05_field_data/), containing detailed sampling event information including garden ID, anonymised observer ID, sampling times, weather conditions, and field comments (raw_sampling_data.xlsx),
• trait data (06_trait_data/), listing morphological trait measurements for each captured insect individual, including intertegular distance, proboscis length, and other traits relevant to pollination (individual_traits.csv). The distribution of selected trait values is visualised in Fig.3. We additionally record whether the interaction was pollination or nectar robbery (individual_traits.csv, see Table 2). We also provide summarised species data per garden, date and hourly sampling interval (see Fig.2 for some examples) as an abundance matrix (species_temporal_flower_visitation_matrix.csv),
• pollination success data (07_pollination_success/), containing seed or fruit set data per garden and per individual plant. Separate CSV files are provided for each plant species. For *D. carota,* we measured seed set. For *R. sativus* and *S. officinale,* both seed and fruit set were measured. For *O. viciifolia,* fruit set was measured. The distribution of data in each of the six datasets are presented in Fig. 4, and
• scripts (08_scripts/), providing R scripts reproducing Fig. 1, Fig. 2, Fig. 3, Fig. 4 and Table 2 in this paper (e.g. to reproduce Fig. 1, see figure_1.R). Please note that we do not provide code to reproduce the map in Fig. 5 as the maps are publicly available.

**Fig. 1:**
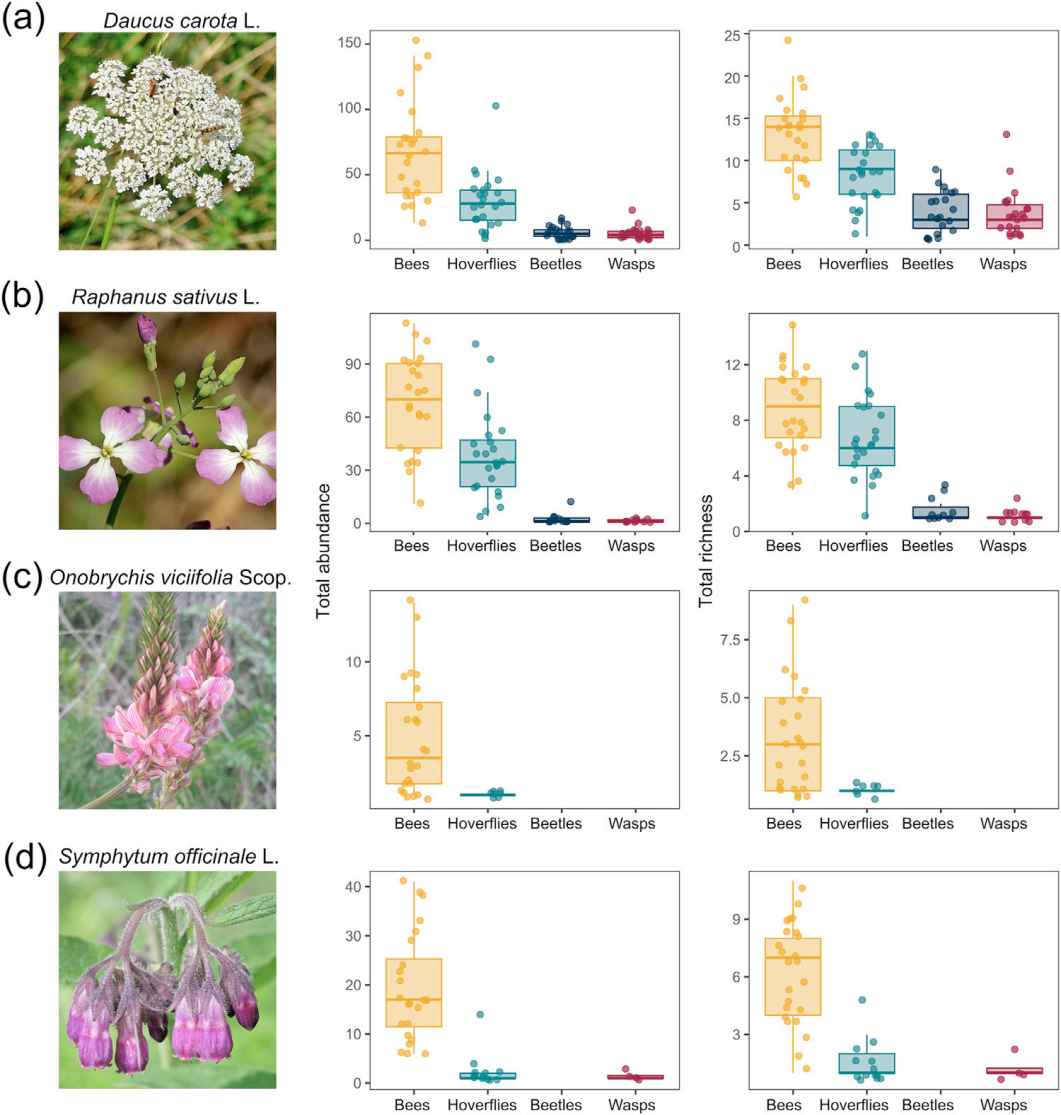
The abundance and richness of pollinator groups per phytometer species. Presented is a photo of the phytometer species on the left, followed by boxplots of pollinator abundances and taxonomic richness, pooled across all sampling intervals per garden. The four phytometer plant species used in this study, are: a) *Daucus carota* (Photo: Konrad Lackerbeck), b) *Raphanus sativus* (Photo: Alan Schmierer), c) *Onobrychis viciifolia* (Photo: Javier Martin), and d) *Symphytum officinale* (Photo: Robert Flogaus-Faust). All photos are sourced via Wikimedia Commons (CC-BY). Where box plots are fully missing, the pollinator group was never observed on the plant species (e.g. beetles on *S. officinale*).

**Fig. 2:**
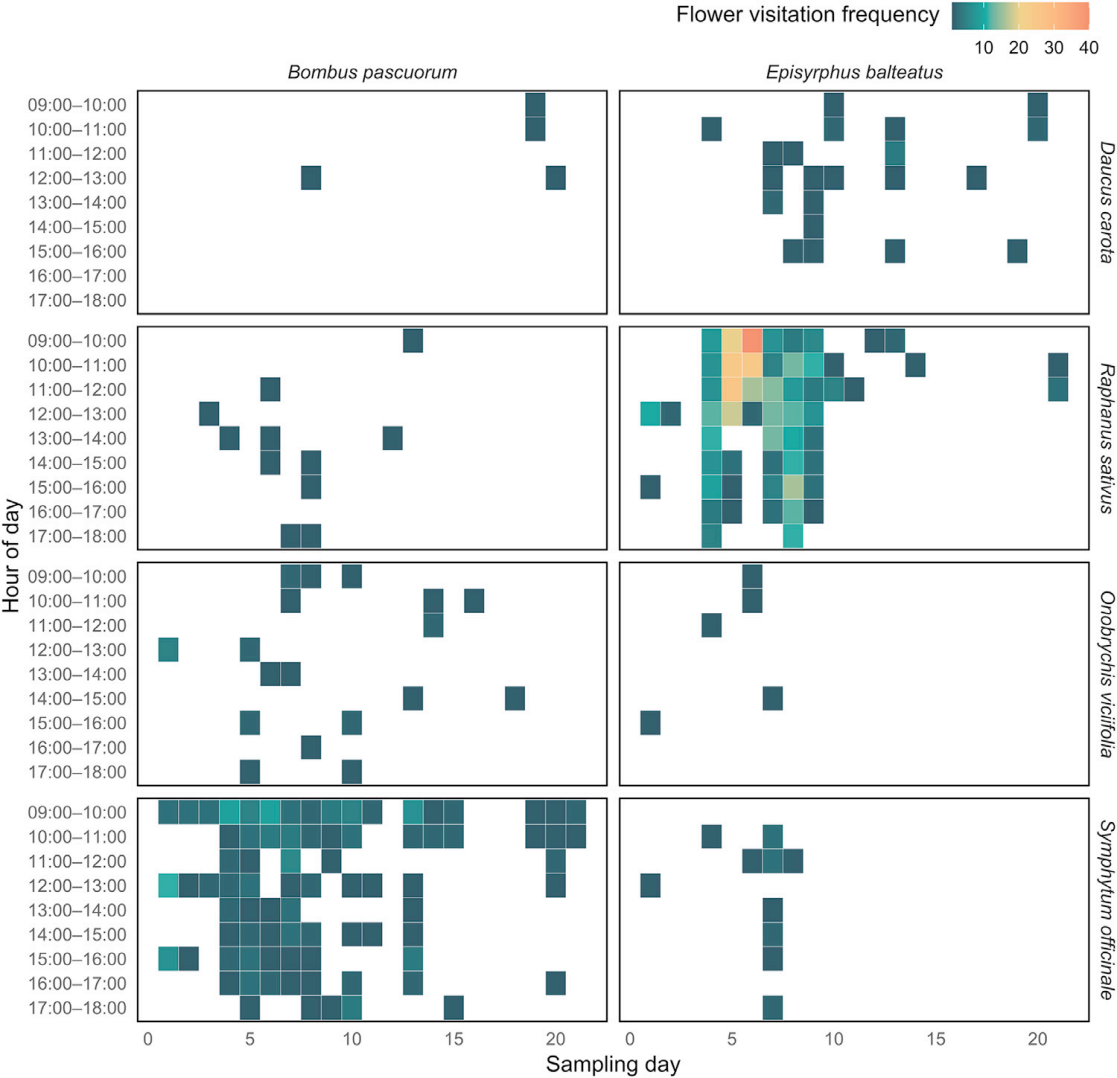
Hourly flower visitation patterns of two example pollinator species across four phytometer plant species. Each tile represents the number of individual visits recorded by *Bombus pascuorum* (left column) and *Episyrphus balteatus* (right column) on each sampling day (x-axis) and hourly time window (y-axis), pooled across all gardens. The colours indicate the flower visitation frequency (i.e., number of individuals captured), cooler colours represent lower values while warmer colours represent larger values.

**Fig. 3:**
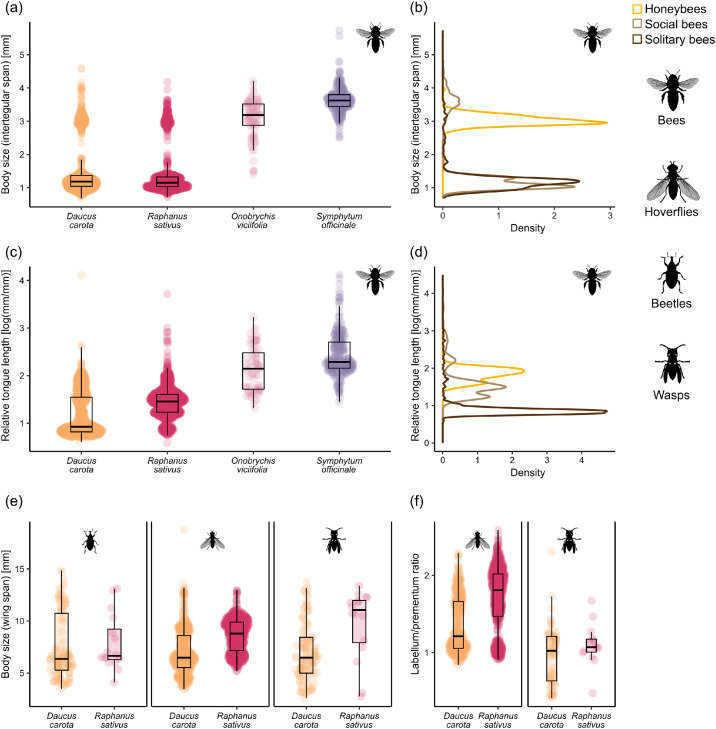
Visualisation of the distribution of selected trait values of pollinator individuals. Presented are body size (a-b and e), relative tongue length (c-d) and tongue shape (f), for bees (a-d) and non-bee pollinators (e-f) and per phytometer species. Depicted are violin plots, which represent the density distribution of each trait in combination with boxplots. The hoverfly and bee icons are accredited to Melissa Broussard, the wasp icon to Andy Wilson, and the beetle icon to Dorota Paczesniak, made freely available from Phylopic.org.

**Fig. 4:**
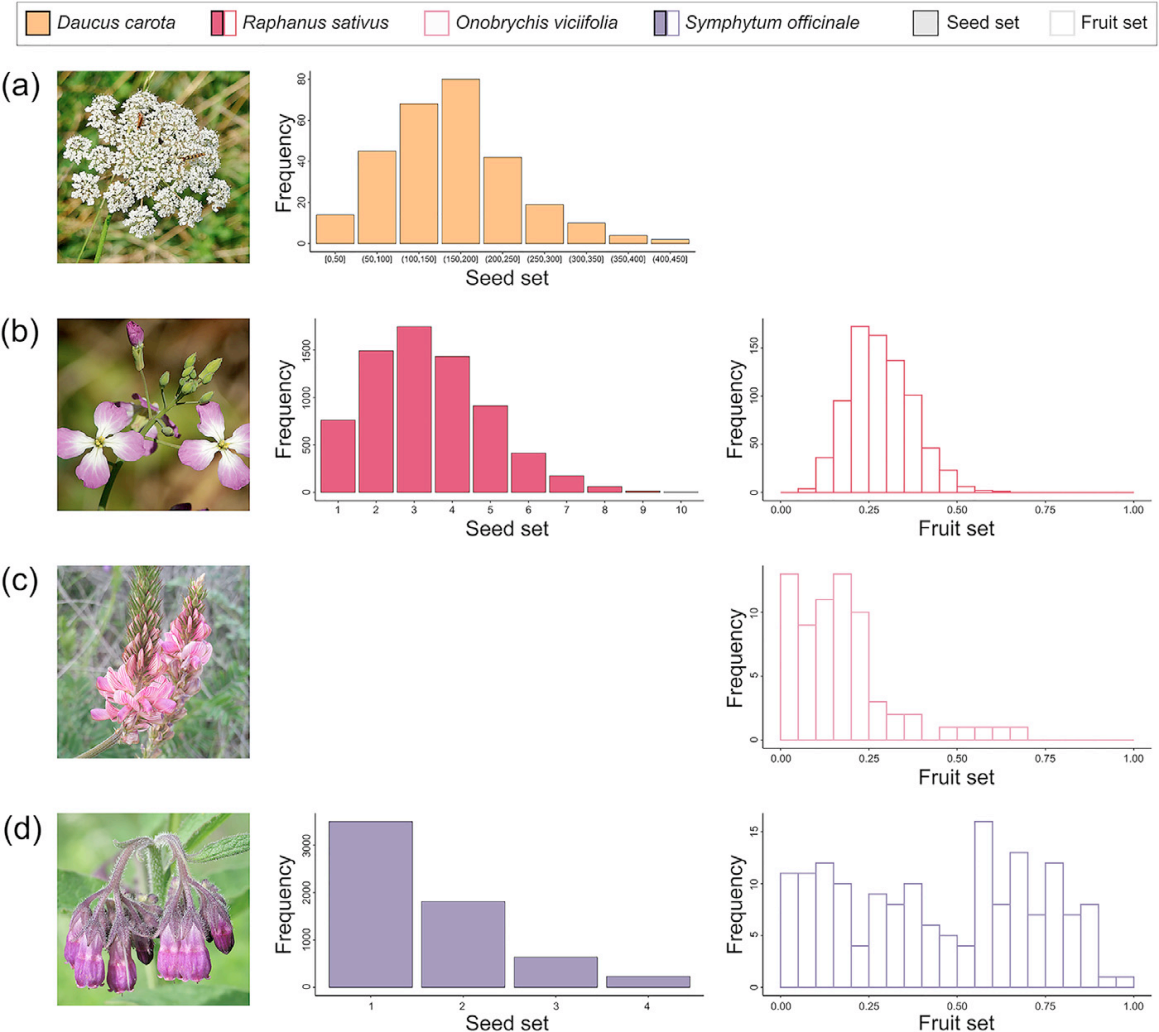
Pollination success data for the four phytometer species. Presented are histograms of fruit and or seed sets for each phytometer: *Daucus carota* (a), *Raphanus sativus* (B) *Onobrychis viciifolia* (c), and *Symphytum officinale* (d). Note that for (a) each bar represents a 50-point range, e.g. [0,50] represents values from 0 to 50. Columns with colourful fills and a black outline represent seed set data, while columns with white fills and a colourful outline represent fruit set data. Fruit set data are not shown for *Daucus carota*, as we counted seeds per umbel and did not assess fruit set. For *Onobychis viciifolia*, seed set is shown in place of fruit set because each pod contains only one seed, and unfertilized pods do not develop. The photos for the plants are sourced as follows: *Daucus carota* (Konrad Lackerbeck), *Raphanus sativus* (Alan Schmierer), *Onobrychis viciifolia* (Javier Martin), and *Symphytum officinale* (Robert Flogaus-Faust). All photos are sourced via Wikimedia Commons (CC-BY).

**Fig. 5:**
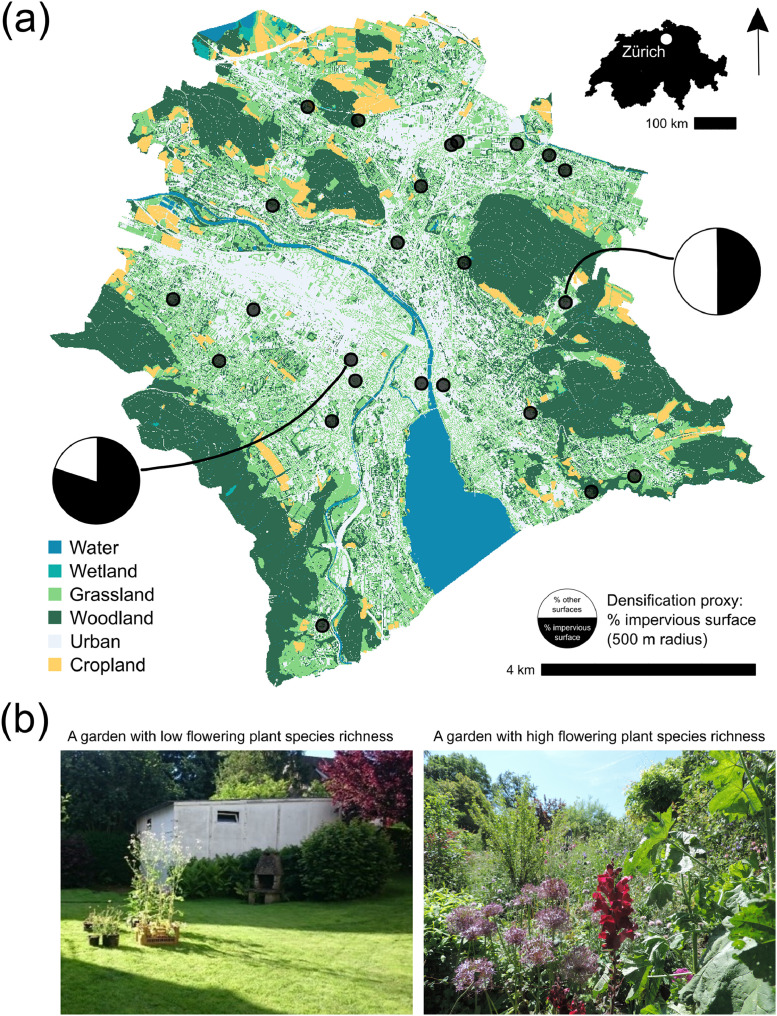
Study design. A map of the 24 gardens sampled in the city of Zurich, Switzerland (a), the black points represent the sites. Gardens were selected along two independent gradients: densification in cities (the proportion of impervious cover in 500-m radius around each garden) and local flowering plant species richness. Examples of gardens with low and high local flowering plant species richness are presented (b). The Habitat Map of Switzerland [17] was used as a base for this map. The political boundaries of the city of Zurich were defined by swissBOUNDARIES3D [18].

**Table 1:** Data structure.

Folder	File name	File type	Description	Records	Variables
01_metadata/	data_description.xlsx	Excel spreadsheet	Column descriptions for all tabular datasets, each dataset has its own sheet	–	–
01_metadata/	README.txt	Text file	READ ME text file summarising the structure of the data in this repository	–	–
02_sampling_protocol/	protocol_german.pdf	PDF	Sampling protocol (original, in German)	–	–
02_sampling_protocol/	protocol_english.pdf	PDF	Sampling protocol (translated into English)	–	–
03_site_data/	garden_site_coordinates.csv	Comma Separated Values	Garden identity and geographic coordinates, in latitude and longitude	24	3
04_taxonomic_data/	taxa_checklist.csv	Comma Separated Values	Taxonomic information for all recorded taxa (e.g. order name, family name, taxon, rank of identified taxon)	168	7
05_field_data/	raw_sampling_data.xlsx	Excel spreadsheet	Raw sampling data including garden identity, anonymised observer ID, capture time period, sampling effort in minutes, wind speed and cloud cover per capture time period, the number and type of escaped invertebrates per phytometer species, number of flowers or umbels per plant, and additional field comments	671	21
06_trait_data/	individual_traits.csv	Comma Separated Values	For each individual, the garden identity, capture data and time window, phytometer plant species, sex, and measured traits are provided	5,795	18
06_trait_data/	species_temporal_flower_visitation_matrix.csv	Comma Separated Values	For each garden, phytometer plant and hourly interval, and pollinator species, the flower visitation frequency per hour is provided as a matrix.	21,120	171
07_pollination_success/	daucus_carota_seed_set.csv	Comma Separated Values	*Daucus carota* seed set, presented as number of seeds per garden, and plant	284	9
07_pollination_success/	raphanus_sativus_fruit_set	Comma Separated Values	*Raphanus sativus* fruit set, presented as number of flower with and without fruits per garden and plant	787	8
07_pollination_success/	raphanus_sativus_seed_set	Comma Separated Values	*Raphanus sativus* seed set, presented as number of seeds per garden, and plant	7,076	8
07_pollination_success/	onobrychis_viciifolia_fruit_set	Comma Separated Values	*Onobrychis viciifolia* fruit set, presented as number of flower with and without fruits per garden and plant	72	8
07_pollination_success/	symphytum_officinale_fruit_set.csv	Comma Separated Values	*Symphytum officinale* fruit set, presented as number of flower with and without fruits per garden and plant	163	9
07_pollination_success/	symphytum_officinale_seed_set.csv	Comma Separated Values	*Symphytum officinale* seed set, presented as number of seeds per garden, and plant	6,181	10
08_scripts/	figure_1.R	R script	Script to reproduce Fig. 1	–	–
08_scripts/	figure_2.R	R script	Script to reproduce Fig. 2	–	–
08_scripts/	figure_3.R	R script	Script to reproduce Fig. 3	–	–
08_scripts/	figure_4.R	R script	Script to reproduce Fig. 4	–	–
08_scripts/	table_2.R	R script	Script to reproduce Table 2	–	–

## 4 Experimental Design, Materials and Methods

We selected 24 home gardens (mean area ± SD: 396.18 ± 153.40 m^2^) to vary independently in their amount of local flowering species richness and level of urban densification (the proportion of impervious cover in 500-m radius around each garden). Suitable gardens were identified based on the urban habitat map of the city of Zurich (Fig. 5a) and during field prospections [11]. All gardens were open, sunny sites with at least 7–9 hours of daily sun exposure during the experiment. They represent a subset of gardens from the BetterGardens project, and further details on the floral richness and impervious surface cover can be accessed in the associated dataset [4].

### Phytometer species

We used a phytometer species approach with the following four insect-pollinated plant species: wild carrot (*Daucus carota* L.; five pots), radish (*Raphanus sativus* L.; six pots), common sainfoin (*Onobrychis viciifolia* Scop.; five pots) and common comfrey (*Symphytum officinale* L.; three pots). These species have an out-crossing mating system, either by being self-incompatible or due to a flower morphology preventing self-pollination. Thus, seed and or fruit set (direct measures of plant reproductive success) should largely depend on pollen transfer and pollination service provided by pollinators [77]. The four phytometer species were selected based on their expected variation in flower visitor specificity because of their differences in floral types (i.e. access to nectar [12]): (a) a flower with exposed nectar (“halophilous”), *D. carota*, (b) a flower with partially concealed nectar (“hemiphilous”), *R. sativus*, (c) a flower with concealed nectar (“euphilous”), *O. viciifolia*, and (d) a flower with deeply concealed nectar (“euphilous”), *S. officinale*. Phytometer species were also chosen for their large numbers of flowers (>100) or inflorescences per plant, similar plant height (approx. 30-100 cm) and a long and overlapping flowering time (May-August).

Seeds of *R. sativus* were sown on March 9, 2016, into 1.5 L pots, which were filled with commercial standard garden soil and placed in a greenhouse. They were transferred to 7.5 L pots on May 22. *S. officinale, O. viciifolia* and *D. carota* were bought as potted plants from certified Swiss wild-flower nurseries (P. Willi, 6274 Eschenbach and UFA Samen, 8408 Winterthur) in March 2016. They were transferred into 10 L pots between 20th and 25 April 2016. *O. viciifolia* plants were grown together in one 20 L pot due to their relatively small size. All plants were kept outdoors under cool conditions to harden them from the end of March onwards. All potted plants were transferred to focal gardens on the same day (June 9, 2016) at the onset of flowering. In the gardens the plants were watered at least weekly and more if necessary. An array of 19 pots of four plant species was set up in the centre of each garden (e.g. Fig. 5b) for a total of 456 experimental pots. Flower or inflorescence abundance was counted during each pollinator observation round by individually counting all flowers in all phytometer species except for *D. carota*, where umbels were counted.

### Flower visitor frequency and species richness

We recruited and trained 37 volunteers, so that we were able to sample up to nine gardens per day. Volunteers were randomly allocated to gardens for each sampling round but were never assigned the same garden more than once. Flower-visiting insects were sampled on each individual of the four phytometer species during their peak flowering time between June 15 and July 20, 2016. We recorded cloudiness on the okta scale, which ranges from 0 (cloudless) to 9 (sky obstructed from view). No fieldwork was conducted on days with okta scale values higher than 6 (overcast sky). We recorded the wind speed on a four-point Beaufort (Bft) scale from 0 (calm) to 12 (hurricane-force). No fieldwork was conducted at wind conditions above 3 Bft (gentle breeze). In each garden, flower visitors were sampled by one to three volunteers simultaneously for nine full and consecutive hours between 9:00 to 18:00 h. Each sampling round was repeated at least three times in each garden. This enabled us to determine hourly flower visitation frequency per insect species (or flower visitor group, respectively) during each of the nine consecutive hours during each sampling day.

Insects were collected after landing on an open flower using a 50 mm by 100 mm polypropylene beaker with a foam plug (Semadeni AG, Ostermundigen). Each insect individual was transferred under a sweep net from the tube to an 8 ml glass tube, which was labelled with the respective phytometer plant and capturing time window and put on cooling elements within cooling bags. Flower visitors were transferred to the lab after each observation round and kept under -20°C until determination by taxonomic experts (see: Acknowledgements). The four most abundant flower visitor taxa: bees (Hymenoptera: Anthophila), hoverflies (Syrphidae), wasps (several clades) and beetles (Coleoptera: several families) were determined to species level, sexed and re-transferred to -20°C immediately after identification.

We had additionally identified some flower visitors in the field to be illegitimate pollinators, for example, large bees accessing nectar through self-bitten holes in the corolla tube (i.e. nectar robbing) or small bees or hoverflies crawling on corollas of *S. officinale* and *O. viciifolia* without touching reproductive parts of the flower. This information was also recorded with the individual data.

### Measurement of pollinator functional traits

Body size and tongue length of all sampled bee and hoverfly individuals were measured. In bees, body size was measured as intertegular distance [13] and tongue length was measured as the sum of the lengths of prementum and the glossa with an Olympus SZX12 Microscope and Olympus image analysis software (Version 510; Olympus Soft Imaging Solutions GmbH). In hoverflies, body size was measured as wingspan, and tongue length was measured as the sum of the lengths of labellum, prementum and fulcrum following Gilbert (1981) [14] with the software ImageJ 1.x[15] based on microphotographs made with a Leica stereo microscope. Since medium-tongued hoverfly species were very rare and long-tongued hoverflies (e.g. *Rhingia* spp.) lacking altogether, the labellum/ prementum ratio (i.e. proboscis shape) was preferred over tongue length [14,16].

### Measurement of plant reproductive success

All plants were collected after the end of flowering—between August 3 and 4, 2016. Flowers produced after the end of the experiment were marked and excluded from the analyses. Fruits and seeds were left to mature in the greenhouse. Fruit set, which is the proportion of successfully fertilised flowers, and/or seed set, which is the number of seeds, were determined for all plants before September 5 according to the following protocol: On each *D. carota* plant, seed set was determined by counting all seeds produced on 20 randomly drawn umbellets of the primary umbel and of all major secondarily produced umbels. In *R. sativus*, entire plants were assessed, and the number of flowers that developed fruits and the number of flowers that did not develop fruits were counted. Additionally, on each plant, the number of seeds was counted in 50 undamaged, randomly drawn fruits. In *O. viciifolia*, entire plants were assessed. We counted both the number of flowers that developed and did not develop fruits. In *S. officinale*, reproductive success was assessed on entire plants. The number of flowers that developed fruits and the number of flowers that did not develop fruits were counted for each branch. Additionally, we counted the number of seeds per fertilised flower. Seed and/or fruit set of 108 *D. carota*, 144 *R. sativus*, 72 *O. viciifolia* and 72 *S. officinale* plants could be successfully measured. We assessed 271 seeds in *D. carota*, 751 fruits and 6,716 seeds in *R. sativus*, 62 fruits in *O. viciifolia*, and 157 fruits and 6,716 seeds in *S. officinale*, from 456 pots.

## Limitations

*O. viciifolia* flowered earlier than the other species and was no longer blooming during some sampling rounds. In analyses including *O. viciifolia*, gardens with too few data points should be excluded, see [1]. Additionally, some flower-visiting insects escaped after capture but before trait measurements. These incidents are recorded in the raw data and result in occasional gaps in the trait dataset. The individuals are still included in the trait dataset so they may be included in analyses considering visitation frequency.

While our design used four standardised phytometer plants across gardens, we did not quantify pollinator visitation to co-flowering garden plants. With 54 to 150 insect-pollinated species flowering per garden, tracking hourly flower visitation frequencies for all pollinators on all plant species was infeasible. We acknowledge that this provides an incomplete composition of the garden-wide pollinator community, and consequential biases in measured flower visitation frequencies. For example, observers occasionally noted high pollinator activity on background plant species such as lavender, suggesting that some pollinators may have ignored the experimental plants in favour of such background plants. However, the pollinator community had been sampled with trap nests [19] in parallel to the study. Moreover, a comprehensive sampling of flower-visiting insects was conducted the previous year using coloured bowl traps [20]. As such, community data can still be interpolated.

## Ethics Statement

The authors confirm that they have read and followed the ethical requirements for publication in *Data in Brief*. This work did not involve human subjects, animal experiments, or data collected from social media platforms. Invertebrate sampling was conducted in 24 privately-owned home gardens with landowner permission. According to Swiss legislation, no special permits were required, as sampling did not involve protected areas or protected species.

## CRediT Author Statement

Merin Reji Chacko: Data curation, Formal analysis, Writing - original draft, Writing - review and editing. David Frey: Conceptualization, Methodology, Resources, Data curation, Investigation, Project administration, Funding acquisition, Writing - original draft, Writing - review and editing. Marco Moretti: Conceptualization, Methodology, Resources, Investigation, Writing - Reviewing and Editing, Supervision, Project administration, Funding acquisition.

## Data Availability

EnviDatComprehensive dataset of pollinator diversity and visitation rates with individual-based traits and pollination success across four urban garden plant species (Original data)
